# Physical activity improves the visual–spatial working memory of individuals with mild cognitive impairment or Alzheimer’s disease: a systematic review and network meta-analysis

**DOI:** 10.3389/fpubh.2024.1365589

**Published:** 2024-03-28

**Authors:** Jie Deng, Hong Wang, Tingting Fu, Chong Xu, Qiqi Zhu, Liya Guo, Yu Zhu

**Affiliations:** ^1^College of Physical Education, Southwest University, Chongqing, China; ^2^College of Physical Education and Health Sciences, Chongqing Normal University, Chongqing, China; ^3^Ministry of Sports and National Defense Education, Chongqing College of Electronic Engineering, Chongqing, China

**Keywords:** mild cognitive impairment, Alzheimer’s disease, visual–spatial working memory, physical exercise, network meta-analysis

## Abstract

**Objective:**

Our network meta-analysis aimed to ascertain the effect of physical activity on the visual–spatial working memory of individuals with mild cognitive impairment and Alzheimer’s disease as well as to propose tailored exercise interventions for each group.

**Methods:**

Employing a frequentist approach, we performed a network meta-analysis to compare the effectiveness of different exercise interventions in improving the visual–spatial working memory of individuals with mild cognitive impairment and Alzheimer’s disease. Subsequently, we explored the moderating variables influencing the effectiveness of the exercise interventions through a subgroup analysis.

**Results:**

We included 34 articles involving 3,074 participants in the meta-analysis, comprised of 1,537 participants from studies on mild cognitive impairment and 1,537 participants from studies on Alzheimer’s disease. The articles included exhibited an average quality score of 6.6 (score studies) and 6.75 (reaction time [RT] studies), all passing the inconsistency test (*p* > 0.05). In the mild cognitive impairment literature, mind–body exercise emerged as the most effective exercise intervention (SMD = 0.61, 95% CI: 0.07–1.14). In Alzheimer’s disease research, aerobic exercise was identified as the optimal exercise intervention (SMD = 0.39, 95% CI: 0.06–0.71).

**Conclusion:**

The results of the subgroup analysis suggest that the most effective approach to enhancing the visual–spatial working memory of individuals with mild cognitive impairment entails exercising at a frequency of three or more times per week for over 60 min each time and at a moderate intensity for more than 3 months. Suitable exercise options include mind–body exercise, multicomponent exercise, resistance exercise, and aerobic exercise. For individuals with Alzheimer’s disease, we recommend moderately intense exercise twice per week for over 90 min per session and for a duration of 3 months or longer, with exercise options encompassing aerobic exercise and resistance exercise.

## 1 Introduction

Baddeley and Hitch (1) have emphasized a central theoretical concept of working memory, which is that working memory is a cognitive mechanism that retains a limited amount of information during an active state to utilize the information in an ongoing task. This definition highlights the role of working memory in a wide range of behaviors and tasks (1). Baddeley has expanded upon the original three-component model proposed in 1974 by introducing an additional subsystem known as the episodic buffer, thereby proposing a comprehensive four-component model of working memory. This extended model comprises the central executive system, visual–spatial sketchpad, episodic buffer, and phonological loop. Positioned at the highest level, the central executive system orchestrates the most intricate executive functions, while the visual–spatial sketchpad, episodic buffer, and phonological loop occupy the second tier, primarily responsible for the transient manipulation of information. Notably, the four-component model underscores the interconnectedness between the phonological loop and visual–spatial sketchpad (2).

The concept of visual–spatial working memory (VSWM) has been proposed by Logie in 1995 and is an important component of working memory, involving the storage and manipulation of spatial information in short-term memory (3). VSWM consists of two elements, visual memory and spatial memory, and is mainly used to temporarily store visual–spatial information (4). VSWM memorizes figures or shapes, and spatial working memory refers to the memory of spatial orientation information and presentation order of objects (5). Simultaneously, research indicates that, compared to the phonological loop, VSWM relies more on central executive functions. This may be a significant factor in visual–spatial working memory, serving as a prominent indicator of cognitive decline.

Mild cognitive impairment (MCI) represents an initial phase of cognitive deterioration characterized by mild deviations in memory, attention, language, and executive functions. Under MCI, these deviations have not yet progressed to a severe level and do not disrupt an individual’s capacity to carry out activities of daily living (6). MCI is characterized by a higher risk of cognitive impairment compared to healthy peers and serves as a crucial early warning signal for the development of Alzheimer’s disease (AD) (6). However, the conversion rate of MCI to AD varies. Follow-up studies have shown that within two to three years, the conversion rate to AD can increase to 50% (7). In a study comparing the VSWM among individuals with MCI and AD and healthy individuals, it has been found that one-fifth of healthy older adults, one-third of individuals with MCI, and half of individuals with AD exhibited visual–spatial working memory impairments (8). They may experience slower memory, lower accuracy, and shorter durations for processing and manipulating spatial information (9). A study on the VSWM of individuals with MCI has found that they display impaired encoding and recognition functions in VSWM tasks, indicating that they may need to exert more effort to maintain their cognitive status (9). Thus, it is necessary to focus on and improve the VSWM of individuals with MCI.

AD is a progressive neurodegenerative disorder and ranks among the most prevalent causes of dementia. The main features of AD are memory loss and a progressive decline in other cognitive functions, including attention, language, spatial orientation, and executive functioning (10). VSWM is mostly associated with the brain’s dorsal pathway, which consists of three pathways that project from the parietal regions to the prefrontal cortex, premotor cortex, and medial temporal lobe regions (11). A disconnection between the ventral and dorsal pathways has been identified in individuals with AD and may potentially play a role in the observed deficits in the VSWM among these individuals (12). Impaired VSWM function resulting from AD may lead to a loss of spatial orientation, an inability to differentiate directions, and even disorientation in familiar environments. As the disease progresses, AD can lead to behavioral and personality changes that affect all aspects of daily life.

Existing research has elucidated the neurophysiological mechanisms underlying the enhancement of working memory in individuals with cognitive impairment through exercise. Specifically, the augmentation of hypothalamic volume through physical exercise has been identified as a significant contributor to memory improvement and the reversal of age-related shrinkage in hypothalamic volume (13). Moreover, physical exercise has been shown to stimulate the generation of new nerve cells, and aerobic exercise induces the expansion of brain tissue, leading to increased gray and white matter in crucial cognitive regions, such as the prefrontal cortex (14, 15). This collective evidence underscores the multifaceted impact of physical exercise on cognitive function in individuals with cognitive impairment, offering insights into its neurobiological underpinnings.

Previous research has confirmed the beneficial effects of physical exercise on the VSWM of individuals with MCI and AD (16–19). Relevant meta-analyzes and experimental studies have discussed overall cognitive function, working memory, and executive function (20, 21). These studies’ intervention programs include aerobic (22), resistance (23), multicomponent (24), and mind–body exercises (25), and different intensities (low-intensity, moderate-intensity, and high-intensity) (26) have been examined. Additionally, these studies examine the impact of exercise on the VSWM of healthy individuals (27), with particular emphasis on its effects in children (28). However, research on the effects of exercise interventions on the VSWM of individuals with different stages of cognitive impairment is limited. As an important marker of cognitive decline, VSWM needs to be monitored and improved at different stages of cognitive impairment. Does exercise produce improvements in VSWM of individuals with different stages of cognitive impairment? Which physical exercise programs offer the best improvement of VSWM in individuals with MCI and AD? These questions are the focus of our study.

Therefore, our systematic review aims to investigate the impact of exercise on VSWM in individuals with MCI and AD separately. Additionally, a comprehensive net meta-analysis will be undertaken, grounded in the initial meta-analysis, to furnish quantitative evidence regarding the enhancement of the VSWM of individuals with MCI and AD. This will be achieved by directly or succinctly contrasting various physical activity regimens through the net meta-analysis, thereby facilitating the visualization of comparative outcomes (29). Furthermore, the optimal physical activity regimen will be discerned through a systematic ranking process, contributing to a refined understanding of the potential benefits of exercise on the VSWM of individuals with MCI and AD.

## 2 Materials and methods

We have registered the protocol for this systematic review on PROSPERO (CRD42023459625). The network meta-analysis followed the PRISMA extension statement for systematic reviews reporting on network meta-analyzes (30).

### 2.1 Eligibility criteria

Two authors (JD and TTF) conducted the initial screening of studies. Our meta-analysis included studies meeting the following criteria: (1) Participants had been diagnosed with MCI or AD, regardless of age. (2) Participants had either normal vision or vision corrected through aids. (3) The intervention involved a distinct form of physical activity, satisfying two conditions: skeletal muscle movement and energy expenditure. (4) All outcomes were quantified using validated measurement tools, encompassing both voluntary physical and mental strength. (5) We exclusively considered randomized controlled trials (RCTs) due to their providing the highest level of evidence in meta-analyzes. We excluded reports that did not meet the following criteria: (1) The publication was not in English or Chinese. (2) The full text or essential data (such as means and standard deviations) were unavailable.

### 2.2 Information sources

We conducted a prospective search on September 15, 2023, utilizing WoS, MEDLINE, BIOSIS Previews, PubMed, the China National Knowledge Infrastructure (CNKI), and Wanfang Data (Chinese) databases to find relevant papers. Due to limited human resources, only six databases with RCTs were chosen for this review. To determine inclusion, we simultaneously reviewed all included study citations. We consulted the study’s corresponding author when needed.

### 2.3 Search strategy

A systematic search strategy was applied using a MeSH word search. For example, the following forms of retrieval were employed: “Mild cognitive impairment” [MeSH] (e.g., “Mild cognitive impairment” OR “Cognitive Dysfunctions” OR “Dysfunction, Cognitive” OR “Cognitive Impairments” OR “Impairment, Cognitive” OR “Cognitive Disorder” OR “Cognitive Disorders” OR “Disorder, Cognitive” OR “Disorders, Cognitive” OR “Mild Cognitive Impairment” OR “Cognitive Impairment, Mild” OR “Cognitive Impairments, Mild” OR “Impairment, Mild Cognitive” OR “Impairments, Mild Cognitive” OR “Isometric Exercises” OR “Mild Cognitive Impairments” OR “Cognitive Decline” OR “Mental Deterioration” OR “Decline, Cognitive” OR “Deterioration, Mental” OR “Deterioration, Mental” OR “Mental Deterioration”) AND “Alzheimer’s disease” [MeSH] (e.g., “Alzheimer Dementia” OR “Alzheimer Dementias” OR “Dementia, Alzheimer” OR “Alzheimer’s Disease” OR “Senile Dementia”) AND “Cognition” [MeSH] (e.g., “Cognitions” OR “Cognitive Function” OR “Cognitive Functions” OR “Function, Cognitive” OR “Functions, Cognitive”) AND “Memory, Short-Term” [MeSH] (e.g., “Memories, Short-Term” OR “Short-Term Memories” OR “Memory, Short term” OR “Memories, Short term” OR “Working Memory” OR “Working Memories” OR “Immediate Memories” OR “Immediate Memory” OR “Memories, Immediate” OR “Recalls, Immediate”).

### 2.4 Study selection

We loaded all main database articles into EndNote to remove duplication. JD and TTF separately retrieved research design, efficacy, and safety data using a structured spreadsheet. If the two reviewers disagreed, a third author decided after reviewing the article. To assure inter-author agreement, JD and TTF individually completed the eligibility evaluation for study inclusion in an unblinded, standardized manner in a pilot test before the official commencement. This allowed us to resolve disagreements by consensus. The main information extracted included (1) basic information, such as author(s) and the year of publication; (2) participant characteristics, such as physical condition (healthy people or those with medical conditions), the mean age or age range of the experimental and control groups, and the sample size; and (3) experimental characteristics such as study design, type of intervention, type of control, information about the intervention (duration of each intervention session and the duration, frequency, and intensity of the intervention), measurement tools, and outcome indicators. One author (TF) extracted important data from the articles’ full texts, abstracts, tables, and charts or Supplementary material. The relevant authors were contacted for missing data for analysis. Second author JD reviewed extracted data.

### 2.5 Risk of bias assessment

Two authors (JD and TTF) conducted independent assessments of the risk of bias within the included studies using the Physical Therapy Evidence Database (PEDro) scale (31). This scale comprises 11 items: eligibility criteria, randomization, concealed allocation, similarity at baseline, blinding of subjects, therapists and assessors, retention rate exceeding 85%, intention-to-treat analysis, between-group comparisons, point measures, and measures of variance. The total quality assessment score, derived from the scores of 10 criteria (excluding the first item, eligibility criteria), ranges from 0 to 10. Per Maher et al. (30) criteria, a score of ≥6 indicates that the assessed study is of high quality, while a score of <6 suggests low quality. Any discrepancies or disagreements between the reviewers were resolved through consultation with a third reviewer (YZ).

### 2.6 Statistical synthesis and analysis

A meta-analysis compared exercise programs to controls. A random-effects model which included a 95% CI and the SMD for each research computed the standardized mean difference (SMD). The I2 statistic with a 95% CI was used to quantify heterogeneity, with thresholds of 0, 25, 50, and 75% indicating absent, low, moderate, and high study inclusion.

The type of exercise and dose parameters may affect the extent of cognitive impact and the duration of these effects post-intervention (32). We explore the optimal exercise regimen based on the FITT principle, which considers frequency, intensity, time, and type (33). Subgroup analyzes were conducted on four moderating variables: exercise intervention intensity was categorized into three levels: low, moderate, and high, supported by research; changes in neurobiological factors induced by exercise may be dose-dependent on exercise intensity (34). The determination of exercise intensity followed the range Borer has proposed, where less than 50% of the maximum oxygen (V02max) consumption is considered low intensity, 50 to 75% is moderate intensity, and more than 75% is high intensity (35). If not explicitly reported in the article, intensity was determined based on principles of physiology and exercise science. The exercise intervention duration was divided into three subgroups: <60 min, 60–89 min, and ≥ 90 min. Although animal model research has suggested that exercise intervention duration influences the corresponding working memory enhancement, its applicability to MCI and AD remains inconclusive (36). Intervention duration was categorized into three groups: <90 days, 90–179 days, and ≥ 180 days. Does a prolonged exercise regimen yield superior effects on VSWM? Is there an optimal effect size? These questions require further subgroup analysis. Weekly intervention frequency was subdivided into three categories: less than twice per week, three to four times per week, and five or more times per week. Is there a correlation between weekly intervention frequency and improvements in MCI and AD through exercise? To answer these questions, an exploration of the optimal intervention frequency is warranted.

A network meta-analysis was conducted using Stata 15.1 within a frequentist framework. The network analysis incorporated the results of each study, including direct comparisons and network evidence from RCTs. We categorize exercise interventions into the following types: aerobic exercise (Walking, cycling, treadmill, etc.), mind–body exercises (combining aerobic exercise with cognitive training, Tai Chi, yoga etc.), multicomponent exercise (involving three or more types of exercise), resistance training (resistance band exercises etc.), acute exercise, and finger exercises. A network graph containing intervention measures at each node was created by comparing each intervention to a common comparator. Lines connecting nodes indicated direct RCT intervention measure comparisons. More research used direct comparisons with thicker lines. The node size indicated how many people received an intervention. Clinical and other factors can cause study heterogeneity, hence a random-effects model was adopted. This method yields conservative CIs.

To evaluate the heterogeneity between the studies, an inconsistency analysis was performed. Inconsistency test *p*-values above 0.05 were analyzed using a consistency model. Exercise interventions were ranked using cumulative ranking curve area (SUCRA) and mean rankings. SUCRA accurately estimates the cumulative ranking probability for top-i treatments.

### 2.7 Risk of publication bias

Our research assessed publication bias by assessing the standard error and its reciprocal for each article. For dispersion visualization, a funnel plot was created. Visual inspection and Egger’s test showed no publication bias because the funnel plot was symmetrical. In order to eliminate publication bias, Begg’s test, an adjusted rank correlation test, was performed.

## 3 Results

### 3.1 Description of the included studies

**Figure 1:**
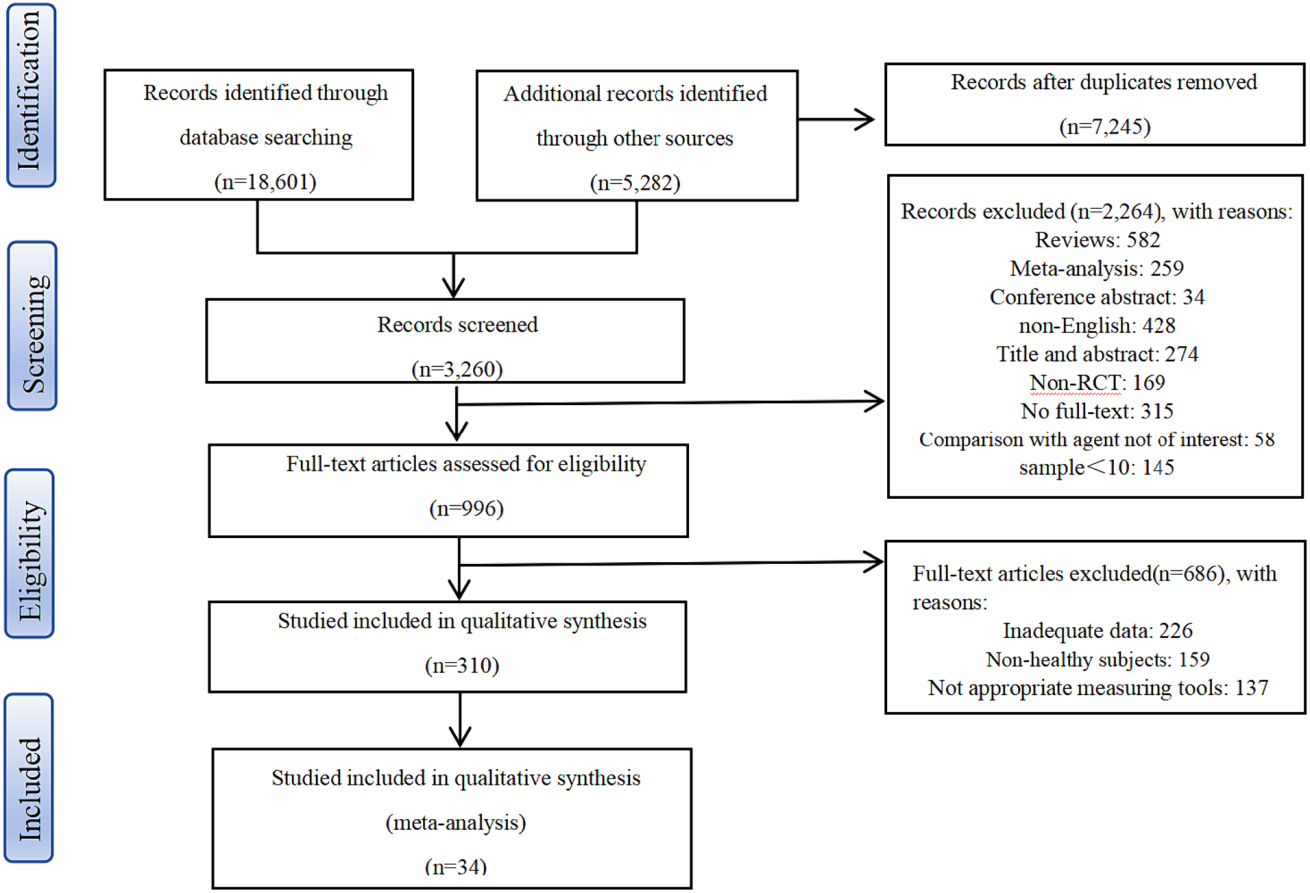
Selection of studios for inclusion.

Initially, 382 articles were included after deleting duplicates and screening titles and abstracts. After applying inclusion and exclusion criteria, this study included 34 articles, 23 of which included MCI individuals and 11 with AD patients. The study involved a total of 3,074 participants, including 1,537 individuals with MCI and 1,537 with AD (Figure 1). Among the 23 MCI studies, there were 27 experiments in total, with eight experiments investigating the effects of aerobic exercise (AE), seven studying the effects of resistance exercise (RE), seven examining the effects of multicomponent exercise (ME), three exploring the effects of mind–body exercise (MBE), one focusing on the effects of finger exercises (FINGER), and one investigating the effects of acute exercise (ACUTE). Notably, five experiments directly compared different exercise interventions, with two employing a three-arm design. Among the 11 AD articles, there were 18 experiments in total, including five exploring the effects of AE, six assessing the effects of RE, and five studying the effects of ME. Two of these experiments directly compared different exercise interventions, both utilizing a three-arm design. Table 1 shows the articles selected for web-based meta-analysis.

**Table 1:** Characteristics of the studies included in the meta-analysis.

Reference	Country	Individual characteristics	Group	Intervention type	Control type	Intervention characteristics	Measuring tool	Outcomes measurement
Yoon et al. (37)	Korea	Older adults with MCI, *n* = 43 Female = 30 (69.8%) EG age: 73.82 ± 4.37 CG age: 74.03 ± 4.27	EG: *n* = 20 CG: *n* = 23	Resistance exercise	DR, S	16 weeks 3 session/week 60 min/session	①②⑥	Score
Gavelin et al. (38)	Sweden	Persons with exhaustion disorder, *n* = 132 Female = 111 (84%) EG-a age: 43.89 ± 9.21 EG-b age: 44.15 ± 8.60 CG age: 41.88 ± 7.41	EG-a: *n* = 44 EG-b: *n* = 47 CG: *n* = 41	Aerobic exercise Cognitive training	NM	12 weeks 3 session/week 40 min/session	①②	Score
Li et al. (39)	Hong Kong, China	Persons with MCI, *n* = 229 Female = 196 (85.6%) EG age: 73.93 ± 7.40 CG age: 74.83 ± 7.57	EG: *n* = 116 CG: *n* = 113	Multicomponent training: Aerobic exercise Resistance exercise Balance exercise Flexibility exercise	NM	8 weeks 3 sessions/week 60 min/session	①②	Score
Eskilsson et al. (40)	Sweden	Persons with exhaustion disorder, *n* = 56 Female = 52 (92%) EG age: 42.00 ± 8.61 CG age: 41.69 ± 7.88	EG: *n* = 24 CG: *n* = 32	Aerobic exercise	CT	12 weeks 3 session/week 40 min/session	①②	Score
Dannhauser et al. (41)	United Kingdom	Persons with MCI, *n* = 67 Female = 28 (42%) Age: 73.9 ± 8.3	NR	Aerobic exercise Cognitive training	NR	12 weeks 3 session/week 30–45 min/session	①②⑥	Score
Devenney et al. (42)	Ireland	Persons with MCI, *n* = 64 Female = 34 (53.2%) EG age: 71.7 ± 5.3 CG age: 69.0 ± 7.2	EG: *n* = 35 CG: *n* = 29	Acute high-intensity Aerobic exercise	NR	12 weeks 90 min/session	⑤	Score
Hong et al. (43)	Korea	Persons with MCI, *n* = 22 Female = 16 (73%) Healthy persons, *n* = 25 Age: 75.53 ± 4.48	EG-MCI: *n* = 10 CG-MCI: *n* = 12 EG-NG: *n* = 12 CG-NG: *n* = 13	Resistance exercise	DR	12 weeks 2 sessions/week 60 min/session	①②	Score
Suzuki et al. (24)	Japan	Older adults with MCI, *n* = 100 Female = 49 (49%) EG age: 74.8 ± 7.47 CG age: 75.8 ± 6.1	EG: *n* = 50 CG: *n* = 50	Aerobic exercise Muscle strength training Postural balance retraining Dual-task training	CT	6 months 2 sessions/week 90 min/session	⑧	Score
Scherder et al. (44)	The Netherlands	Frail older adults with MCI, *n* = 43 Female = 38 (88%) EG-a age: 84 ± 6.38 EG-b age: 89 ± 2.40 CG age: 86 ± 5.05	EG-a: *n* = 15 EG-b: *n* = 13 CG: *n* = 15	Hand/face exercises Aerobic exercise	NM	6 weeks 3 sessions/week 30 min/session	④⑨	Score
Nagamatsu et al. (45)	Canada	Women with subjective memory, *n* = 86 EG-a age: 73.9 ± 3.4 EG-b age: 75.6 ± 3.6 CG age: 75.1 ± 3.6	EG-a: *n* = 28 EG-b: *n* = 30 CG: *n* = 28	Resistance exercise Aerobic exercise	Balance and tone	6 months 2 sessions/week	⑩⑪	Score
Jeong et al. (46)	Korea	Female = 18 (69%) EG age: 70.23 ± 7.47 CG age: 71.77 ± 5.53	EG: *n* = 13 CG: *n* = 13	Aerobic exercise PA promotion Behavior modification Cognitive Exercise multi-task programs	DR	12 weeks 2 sessions/week 90 min/session	⑥	Score
Avenali et al. (47)	Italy	Older adults with amnestic MCI, *n* = 31 Female = 26 (84%) EG age: 72.88 ± 5.60 CG age: 77.29 ± 5.16	EG: *n* = 17 CG: *n* = 14	Aerobic exercises, coordination exercises,	Physical therapy	12 weeks 2 sessions/week 60 min/session	⑥	Score
Suzuki et al. (48)	Japan	Older adults with MCI, *n* = 50 Female = 23 (46%) EG age: 75.3 ± 7.5 CG age: 76.8 ± 6.8	EG: *n* = 25 CG: *n* = 25	Aerobic exercise Muscle strength training Postural balance retraining	CT	12 months 2 sessions/week 90 min/session	⑦⑫	Score
Yang et al. (49)	China	Older adults with non-dementia cognitive impairment after stroke, *n* = 91 Female = 39 (43%) EG age: 74.6 ± 5.5 CG age: 73.4 ± 5.8	EG: *n* = 46 CG: *n* = 45	Resistance training Aerobic training	CT\W	3 months 4 sessions/week 20 min/session	⑰	Score
Wang and Sheng (50)	China	Older adults with MCI, *n* = 94 NR Age: 77.1 ± 4.3	EG: *n* = 46 CG: *n* = 48	Tai Chi	CT	6 months 4 sessions/week 40 min/session	⑥	Score
Lam et al. (51)	Hong Kong, China	Persons with mild dementia, *n* = 376 Female = 301 (80%) EG-a age: 79.8 ± 6.4 EG-b age: 80.3 ± 6.2 EG-c age: 80.7 ± 7.0 CG age: 80.8 ± 6.3	EG-a: *n* = 94 EG-b: *n* = 94 EG-c: *n* = 94 CG: *n* = 94	Working memory training Physical exercise Combined working memory and physical exercise	CT	6 weeks 2 sessions/week 45 min/session	①	Score
van de Rest et al. (52)	The Netherlands	Frail and pre-frail older adults, *n* = 127 Female = 102 (80.3%) Age:≥65 years	EG-a: *n* = 31 CG-a: *n* = 34 EG-b: *n* = 31 CG-b: *n* = 31	Resistance-type exercise training in combination with protein supplementation	Resistance-type exercise	24 weeks 2 sessions/week	①②⑥	Score
Makino et al. (53)	Japan	Older adults with SMC, *n* = 415 Female = 195 (47%) EG-AT age: 72.25 ± 4.56 EG-RT age: 72.33 ± 4.77 EG-CT age: 72.61 ± 4.52 CG age: 72.10 ± 4.61	EG-AT: *n* = 104 EG-RT: *n* = 102 EG-CT: *n* = 104 CG: *n* = 105	Aerobic exercise Resistance exercise Combined exercise	NR	26 weeks 2 sessions/week 60 min/session (a total of 52 sessions)	①	Score
Barnes et al. (54)	United States	Older adults with mild dementia, *n* = 63 Female = 41 (65%) EG age: 64–81.2 CG age: 74.7–82.2	EG: *n* = 32 CG: *n* = 31	Treadmill training	NM	16 weeks 2 sessions/week 30 min/session	①②④⑤⑥	Score
Hoffmann et al. (55)	Denmark	Persons with mild AD, *n* = 200 Female = 87 (43.5%) EG age: 69.8 ± 7.4 CG age: 71.3 ± 7.3	EG: *n* = 107 CG: *n* = 93	Moderate-to-high intensity aerobic exercise	NM	16 weeks 3 sessions/week 60 min/session	⑤	Score
Bossers et al.(56)	The Netherlands	Persons with dementia, *n* = 109 Female = 82 (75%) Age: 85.5 ± 5.1	EG-a: *n* = 37 EG-b: *n* = 36 CG: *n* = 36	Aerobic and strength training	CT\NM	9 weeks 2 sessions/week 30 min/session	⑱	Score
Cheng et al. (57)	Hong Kong, China	Persons with MCI, *n* = 110 Female = 67 (60.9%) EG-m age: 81.9 ± 6.2 EG-t age: 81.8 ± 7.4 CG age: 80.9 ± 7.2	EG-m: *n* = 36 EG-t: *n* = 39 CG: *n* = 35	Mahjong Tai Chi	CT	12 weeks 3 sessions/week	①②	Score
Yu et al. (58)	United States	Older adults with AD dementia, *n* = 96 Female = 43 (45%) EG age: 77.4 ± 6.6 CG age: 77.5 ± 7.1	EG: *n* = 64 CG: *n* = 32	Cycling	S	6 months 3 sessions/week 20–50 min/ session	⑭	Score
Huang et al. (59)	China	Older adults with mild dementia, *n* = 80 Female = 56 (67.5%) EG age: 81.9 ± 6.0 CG age: 81.9 ± 6.1	EG: *n* = 40 CG: *n* = 40	Tai Chi	NM	10 months 3 sessions/week 20 min/session	⑥	Score
Lautenschlager et al. (60)	Australia	Persons who reported memory problems but did not meet criteria for dementia, *n* = 170 Female = 86 (50.5%) EG age: 68.6 ± 8.7 CG age: 68.7 ± 8.5	EG: *n* = 85 CG: *n* = 85	Aerobic exercise	NM	3 sessions/week 50 min/session	④	Score
Cavalcante et al. (61)	Brazil	Older adults with cognitive Female = 47 (62.6%) complaints, *n* = 67 EG-a age: 71 (6) EG-b age: 71 (6) CG age: 71 (4)	EG-a: *n* = 23 EG-b: *n* = 22 CG: *n* = 22	Resistance exercise	CT	12 weeks 3 sessions/week	④	Score
Prick et al. (62)	The Netherlands	Persons with dementia and their family caregivers, *n* = 111 Female = 41 (36.9%) Minimum age of 55 years	EG: *n* = 57 CG: *n* = 54	Flexibility Strengthening Balance Endurance	NM	3 months 3 sessions/week 30 min/session	①②⑮	Score
Sanders et al. (63)	The Netherlands	Persons with dementia, *n* = 91 Female = 43 (62.3%) EG age: 81.7 (7.16) CG age: 82.1 (7.51)	EG: *n* = 39 CG: *n* = 30	Aerobic sessions Lower limb strength exercises	CT\S	24 weeks 3 sessions/week 30 min/session	①②⑥⑦⑯	Score
Pedroso et al. (64)	Brazil	Older adults with diagnosis of AD, *n* = 57 Female = 44 (77.1%) EG-a age: 77.6 ± 6.2 EG-b age: 78.0 ± 5.6 CG age: 79.2 ± 5.6	EG-a: *n* = 22 EG-b: *n* = 21 CG: *n* = 14	Aerobic endurance Flexibility Muscular resistance Balance	NM	12 weeks 3 sessions/week 60 min/session	①②⑥	Score
Stein et al. (65)	Brazil	Persons with AD, *n* = 34 Female = 19 (56%) EG age: 75.27 ± 6.09 CG age: 75.06 ± 6.36	EG: *n* = 18 CG: *n* = 16	Aerobic training	DR	12 weeks 3 sessions/week 60 min/session	⑰	Score
Liao et al. (66)	Taiwan, China	Frail older adults, *n* = 46 Female = 31 (67.3%) EG age: 79.6 (9.0) CG age: 83.8 (5.1)	EG: *n* = 25 CG: *n* = 21	Kinect based exergaming	Combined physical exercise	12 weeks 3 sessions/week 60 min/session	③⑥	RT
Law et al. (67)	Australia	Older adults with MCI, *n* = 83 Female = 50 (60.2%) EG age: 60–85/73.68 (6.8) CG age: 60–88/74.1 (7.6)	EG: *n* = 43 CG: *n* = 40	FcTSim program	CT	13 sessions in 10 weeks	⑥	RT
Thaiyanto et al. (68)	Thailand	Older women with MCI, *n* = 40 EG age: 67.66 ± 2.38 CG age: 68.13 ± 2.65	EG: *n* = 20 CG: *n* = 20	Aerobic exercise Resistance exercise Balance exercise	DR	12 weeks 3 sessions/week 60 min/session	⑥	RT
Law et al. (69)	Australia	Older adults with MCI, *n* = 59 Female = 35 (50.7%) EG-a age: 62–86/76.93 (6.79) EG-b age: 68–88/77.94 (6.11) EG-c age: 64–85/71.57 (7.43) CG age: 60–89/75.14 (8.53)	EG-a: *n* = 15 EG-b: *n* = 16 EG-c: *n* = 14 CG: n = 14	Cognitive training Aerobic exercise	DR	8 weeks 12 sessions more than 40 min	⑥	RT

MCI, mild cognitive impairment; AD, Alzheimer’s disease; NR, Not reported; EG-a: Experimental group a; EG-b, Experimental group b; EG-c, Experimental group c; CG-a, Control group a; CG-b, Control group b, RCT, randomized controlled trial; NM, No movement; CT, Cognitive training; DR, Daily routine; S, Stretching; R, Reading; PC, Passive cycling; W, Walk; ① digits span backward; ② digits span forward; ③ N-back; ④ DS: Digits span; ⑤ Stroop; ⑥ TMT; ⑦Stroop color and word test; ⑧ Wechsler memory scale; ⑨ Visual–spatial working memory; ⑩ Reaction time for spatial memory by a computerized task; ⑪ Accuracy for spatial memory by a computerized task; ⑫ Logical memory subtest of the Wechsler memory scale-revised; ⑬ Memory composite; ⑭ ADAS-Cog; ⑮ 8 words test; ⑯ Visual memory span backward; ⑰ Clock drawing test; ⑱ Visual memory span forward.

### 3.2 Risk of bias

**Figure 2:**
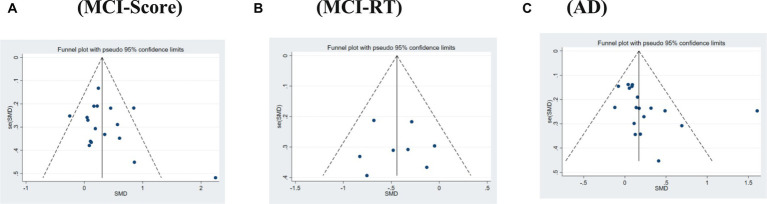
Funnel plot of publication bias. **(A)** MCI-Score. **(B)** MCI-RT. **(C)** AD.

The overall publication bias in the literature for both MCI and AD was evenly distributed in the funnel plot and approximately symmetrical (Figure 2A). When relevant, Begg’s and Egger’s tests were used to assess publication bias in the systematic review, which demonstrated no publication bias overall for both MCI (*p* = 0.769 > 0.1) and AD (*p* = 0.085 > 0.05). Furthermore, in the MCI section, we assessed the publication bias of the studies using scores and reaction time as the outcome measures separately; the results showed no publication bias in either the reaction time studies (*p* = 0.886 > 0.1) or score studies (*p* = 0.238 > 0.1).

### 3.3 Methodological quality evaluation

Quality evaluation of the 34 included articles was conducted utilizing the PEDro scale (Table 2). The mean score for studies using scores as the outcome measure was 6.6, whereas the mean score for studies using reaction time as the outcome measure was 6.75. Furthermore, all articles included in the analysis had conducted “between-group statistical analyzes” and had furnished “point measures and measures of variance.” Of these articles, 30 utilized random assignment for experimental grouping, 28 detailed the process of random assignment, 32 provided information about the baseline levels of participants, 32 presented primary outcome measures for more than 85% of participants, and 32 reported a retention rate and completeness of measurements exceeding 85%.

**Table 2:** PEDro scores and sum of the included studies.

Articles	Item 1	Item 2	Item 3	Item 4	Item 5	Item 6	Item 7	Item 8	Item 9	Item 10	Item 11	Sum (Items 2–11)
*Score*												
Yoon et al. (33)	1	1	0	1	0	0	0	1	1	1	1	6
Gavelin et al. (34)	1	1	0	1	0	0	0	1	1	1	1	6
Li et al. (35)	1	1	0	1	0	0	1	1	1	1	1	7
Eskilsson et al. (36)	1	1	0	1	0	0	0	1	1	1	1	6
Dannhauser et al. (37)	1	1	0	1	0	0	0	0	1	1	1	5
Devenney et al. (38)	1	1	0	1	0	0	0	1	1	1	1	6
Hong et al. (39)	1	1	0	1	0	0	0	1	1	1	1	6
Suzuki et al. (23)	1	1	0	1	0	0	1	1	1	1	1	7
Scherder et al. (40)	1	1	0	1	0	0	1	1	1	1	1	7
Nagamatsu (41)	1	1	1	1	0	0	1	1	1	1	1	8
Jeong et al. (42), Sanders et al. (58)	1	1	0	1	0	0	0	1	1	1	1	6
Avenali et al. (43)	1	1	1	1	0	0	1	1	1	1	1	8
Suzuki et al. (24)	1	1	0	1	0	0	0	1	1	1	1	6
Yang et al. (44)	1	0	0	1	0	0	0	1	1	1	1	5
Wang and Sheng (45)	1	1	0	1	0	0	0	1	1	1	1	6
Lam et al. (46)	1	1	0	1	0	0	0	1	1	1	1	6
van de Rest et al. (47)	1	1	0	1	1	1	0	1	1	1	1	8
Makino et al. (48)	1	1	1	1	0	0	1	1	1	1	1	8
Barnes et al. (49)	1	1	0	1	0	0	1	1	1	1	1	6
Hoffmann et al. (50)	1	1	0	1	1	1	1	1	1	1	1	9
Bossers et al. (51)	1	1	0	1	0	0	0	1	1	1	1	6
Cheng et al. (52)	1	1	0	1	0	0	0	1	1	1	1	6
Yu et al. (53)	1	1	0	1	0	0	0	1	1	1	1	6
Huang et al. (54)	1	1	0	1	0	0	1	1	1	1	1	7
Lautenschlager et al. (55)	1	1	1	1	0	0	1	1	1	1	1	8
Cavalcante et al. (56)	1	1	0	1	1	0	1	1	1	1	1	8
Prick et al. (57)	1	1	1	1	0	1	0	1	1	1	1	8
Sanders et al. (58)	1	0	0	1	0	0	0	1	1	1	1	5
Pedroso et al. (59)	1	1	0	1	0	0	1	1	1	1	1	6
Stein et al. (60)	1	1	0	1	0	0	0	1	1	1	1	6
*Mean (Score)*												6.6
*Reaction time (RT)*												
Liao et al. (61)	1	1	0	1	0	0	1	1	1	1	1	7
Law et al. (62)	1	1	1	1	0	0	1	1	1	1	1	6
Thaiyanto et al. (63)	1	1	0	1	0	0	1	1	1	1	1	7
Law et al. (64)	1	1	0	1	0	0	1	1	1	1	1	7
*Mean (RT)*												6.75

Item 1: eligibility criteria; Item 2: randomization; Item 3: concealed allocation; Item 4: similar baseline; Item 5: blinding of subjects; Item 6: blinding of therapists; Item 7: blinding of assessors; Item 8: more than 85% retention; Item 9: intent-to-treat analysis; Item 10: between-group comparison; Item 11: point measures and measures of variability.

### 3.4 Meta-analysis results

#### 3.4.1 Heterogeneity test

Twenty-three articles report on physical activity’s effect on the VSWM of individuals with MCI, with 15 using scores as the outcome measure and 8 using reaction time. Results for heterogeneity show that the choice of outcome measures may be a source of heterogeneity: I^2^ = 50.6%, *p* < 0.001 for studies related to scores, and I^2^ = 0.00%, *p* < 0.001 for studies related to reaction time (Table 3). The heterogeneous results suggest that other outcome measures may contribute to heterogeneity; hence, independent analyzes were performed for each. Eleven articles examine physical activity’s effect on the VSWM of individuals with AD, using scores as the measure of outcome. The heterogeneity test results showed that I^2^ = 62.9%, *p* < 0.001 (Table 3).

**Table 3:** Heterogeneity analysis of exercise effects of included studies.

		Heterogeneity		Effect size			
		I^2^ (%)	*p*	SMD	95% Cl	Z	*p*
MCI	RT	0	0.492	−0.442	[−0.639, −0.245]	4.39	0.000
	Score	50.6	0.009	0.331	[0.146, 0.516]	3.51	0.000
AD	Score	62.9	0.000	0.23	[0.065, 0.394]	2.74	0.000

MCI, mild cognitive impairment; AD, Alzheimer’s disease; RT, reaction time; SMD, standardized mean difference; CI, confidence interval.

#### 3.4.2 Overall effect size

**Figure 3:**
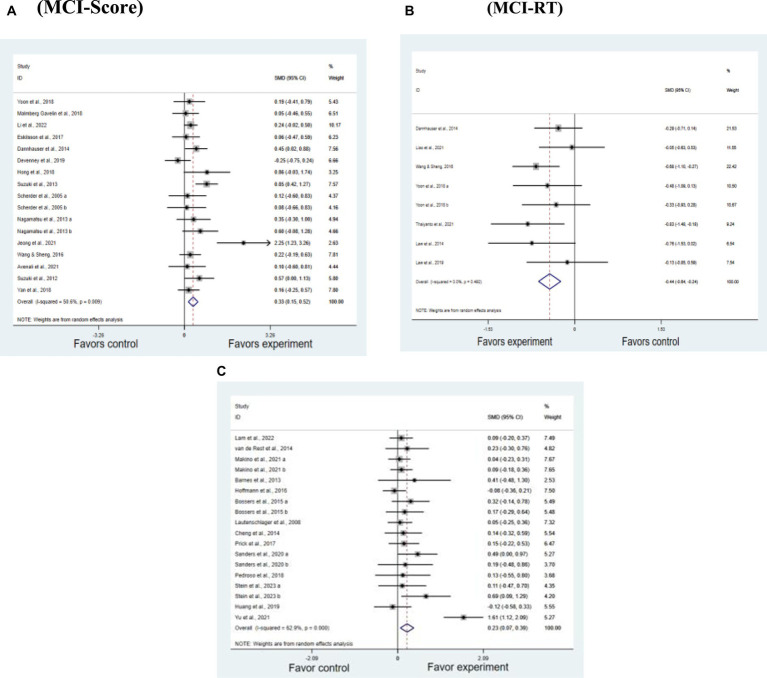
Effect of RT and score outcome measures. **(A)** MCI-Score. **(B)** MCI-RT. **(C)** AD.

As shown by the overall forest plots (Figures 3A–C), both individuals with MCI and AD benefit from physical activity in terms of VSWM. In studies on MCI, the effect size for research related to scores is SMD = 0.331, *p* < 0.05. For studies related to reaction time, the effect size is SMD = −0.442, *p* < 0.05. In AD studies, the effect size is SMD = 0.23, *p* < 0.05 (Table 3).

#### 3.4.3 Subgroup analysis results of MCI studies

In the score group, when the intervention duration was ≥90 min (SMD = 0.875, *p* < 0.05), the intervention effect was slightly better than when it was 60–89 min (SMD = 0.242, *p* < 0.05). The intervention effect for interventions <60 min was not significant. Significant effect sizes were observed when the intervention period was 90–179 days (SMD = 0.226, *p* < 0.05) and ≥ 180 days (SMD = 0.517, *p* < 0.05). When the intervention period was ≤90 days, no significant effect was observed on the improvement of VSWM. Only moderately intense exercise (SMD = 0.455, *p* < 0.05) had a significant intervention effect; exercising three times per week (SMD = 0.591, *p* < 0.05) had a more significant intervention effect on VSWM than exercising for less than two times per week (SMD = 0.213, *p* < 0.05). Exercising five times per week did not produce significant results. Table 4 presents the specific values of the subgroup analysis for MCI regarding score studies.

**Table 4:** Results of MCI subgroup analysis.

Moderator	Subgroup	Type	SMD	95%CI	*Z*	*Q*	*df*	*p*
Intervention duration	<60 min	Score	−0.038	[−0.334, 0.257]	0.25	1.13	3	0.799
	60–89 min	Score	0.242	[0.086, 0.398]	3.04	3.95	8	**0.002**
		RT	−0.494	[−0.752, −0.236]	3.75	4.36	4	**0.000**
	≥90 min	Score	0.875	[0.146, 0.516]	3.26	10.83	3	**0.001**
		RT	−0.34	[−0.671, -0.01]	2.02	1.5	2	**0.044**
Intervention period	<90 days	Score	0.365	[−0.192, 0.922]	1.29	19.05	4	0.199
		RT	−0.426	[−1.037, 0.184]	1.37	1.35	1	0.171
	90-179 days	Score	0.226	[0.026, 0.427]	2.21	4.10	6	**0.027**
		RT	−0.362	[−0.609, −0.114]	2.86	3.38	4	**0.004**
	≥180 days	Score	0.517	[0.267, 0.767]	4.05	4.63	4	**0.000**
		RT	−0.682	[−1.099, −0.266]	3.21	0	0	**0.000**
Exercise intensity level	Low	Score	0.081	[−0.289, 0.45]	0.43	0.02	2	0.669
	Moderate	Score	0.455	[0.207, 0.703]	3.6	24.56	10	**0.000**
		RT	−0.450	[−0.705, −0.196]	3.47	6.26	5	**0.001**
	High	Score	0.139	[−0.294, 0.571]	0.63	4.44	2	0.53
		RT	−0.403	[−0.832, 0.025]	1.84	0.12	1	0.065
Intervention times	≤2 sessions/week	Score	0.591	[0.165, 1.016]	2.72	24.96	7	**0.006**
		RT	−0.426	[−1.037, 0.184]	1.37	1.35	1	0.171
	3–4 sessions/week	Score	0.213	[0.042, 0.384]	2.44	2.15	6	**0.015**
		RT	−0.362	[−0.609, −0.114]	2.86	0	0	**0.004**
	≥5 sessions/week	Score	0.191	[−0.099, 0.482]	1.29	0.04	1	0.197
		RT	−0.682	[−1.099, −0.266]	3.21	1.35	4	**0.001**

RT, reaction time; SMD, standardized mean difference; CI, confidence interval. The bold values are statistically significant.

In the reaction time group, an intervention duration of ≥90 min (SMD = −0.340, *p* < 0.05) had the most significant intervention effect, followed by 60–89 min (SMD = −0.494, *p* < 0.05). Significant effect sizes were observed when the intervention period was ≥180 days (SMD = −0.682, *p* < 0.05) and 90–179 days (SMD = −0.362, *p* < 0.05). No significant improvement in VSWM was observed when the intervention period was ≤90 days. Only moderately intense exercise (SMD = −0.450, *p* < 0.05) had a significant intervention effect. The effect of exercising five times per week (SMD = −0.682, *p* < 0.05) was the most significant, followed by three to five times per week (SMD = −0.362, *p* < 0.05), while exercising less than two times per week was not significant. Table 5 depicts the specific values of the subgroup analysis for reaction time in individuals with MCI.

**Table 5:** Results of AD subgroup analysis.

Moderator	Subgroup	SMD	95% CI	*Z*	*Q*	*df*	*p*
Intervention duration	<60 min	0.148	[−0.063, 0.358]	1.38	2.2	4	0.169
	60–89 min	0.079	[−0.034, 0.191]	1.38	4.23	9	0.169
	≥90 min	0.85	[0.008, 1.693]	1.98	14.76	2	**0.048**
Intervention period	<90 days	0.191	[0.041, 0.340]	2.5	3.87	9	**0.012**
	90–179 days	0.552	[−0.188, 1.291]	1.46	35.07	3	0.144
	≥180 days	0.042	[−0.111, 0.194]	0.54	0.62	3	0.592
Exercise intensity level	Low	0.065	[−0.138, 0.268]	0.63	0.88	2	0.529
	Moderate	0.31	[0.067, 0.554]	2.5	38.31	11	**0.013**
	High	0.167	[−0.188, 0.521]	0.92	4.21	2	0.356
Intervention sessions	1 session/week	0.095	[−0.056, 0.245]	1.23	0.9	4	0.219
	2 sessions/week	0.293	[0.01, 0.576]	2.03	43.05	10	**0.042**
	3 sessions/week	0.245	[−0.082, 0.572]	1.47	0.19	1	0.141

SMD, standardized mean difference; CI, confidence interval. The bold values are statistically significant.

#### 3.4.4 Subgroup analysis results of AD studies

First, the effect of the intervention was found to be significant only when the duration of the intervention was greater than 90 min (SMD = 0.850, *p* < 0.05). Second, a significant effect size was observed only when the intervention period was ≤90 days (SMD = 0.191, *p* < 0.05), whereas there was no significant effect on the improvement of VSWM when the intervention period was 90–179 days or ≥ 180 days. Third, Moderate exercise intensity has an ameliorative effect on VSWM of individuals with AD (SMD = 0.31, *p* < 0.05). Finally, the impact of exercising three times per week (SMD = 0.293, *p* < 0.05) on the intervention effect regarding VSWM was significant, whereas the impact of the remaining two intervention frequencies on the intervention effect was not. Table 5 displays the specific values of the subgroup analysis for AD.

### 3.5 Network meta-analysis results of MCI studies

With the exception of the ACUTE intervention, all categories of exercise interventions demonstrated superior outcomes over the non-exercise control group. In the score group, the results of the inconsistency test indicated no inconsistencies among the different types of physical exercise scores (*χ^2^* = 1.67, *p* = 0.893). The SUCRA ranking shows that MBE ranked the highest, with all five types of physical activity having greater impact rates than the control group; only one type had lower impact rates than the control group. Hence, the SUCRA ranking of the intervention modalities was MBE (82.9), ME (69.8), RE (67.2), AE (57.5), FINGER (42.8), CONTROL (19.0), and ACUTE (10.9), and the differences were statistically significant (*p* < 0.05). A comparison of the different exercise intervention groups with the control group revealed that all exercise interventions, except for FINGER and ACUTE, had a significant effect on VSWM of individuals with MCI, including MBE (SMD = 0.61, 95% CI: 0.07–1.14), ME (SMD = 0.45, 95% CI: 0.04–0.85), RE (SMD = 0.43, 95% CI: 0.07–0.78), and AE (SMD = 0.34, 95% CI: 0.00–0.68).

**Figure 4:**
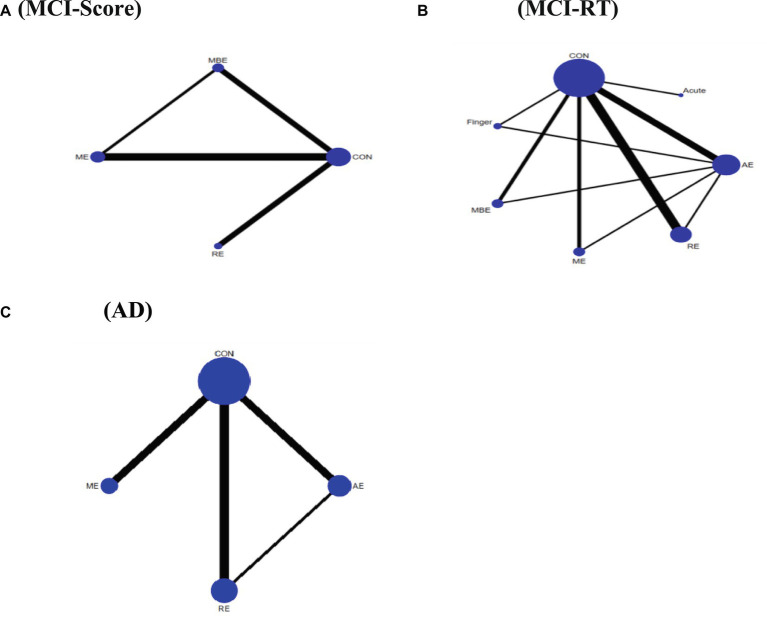
Network meta-analysis of the comparison for different types of exercise interventions. **(A)** MCI-Score. **(B)** MCI-RT. **(C)** AD. Each node represents one intervention, and the connecting lines between the two nodes represent one or more RCTS in which the two interventions are directly compared. The size of each node is proportional to the number of participants randomly assigned, and the thickness of the lines connecting the two nodes is weighted according to the number of RCTS among the interventions that directly compare their connections.

**Figure 5:**
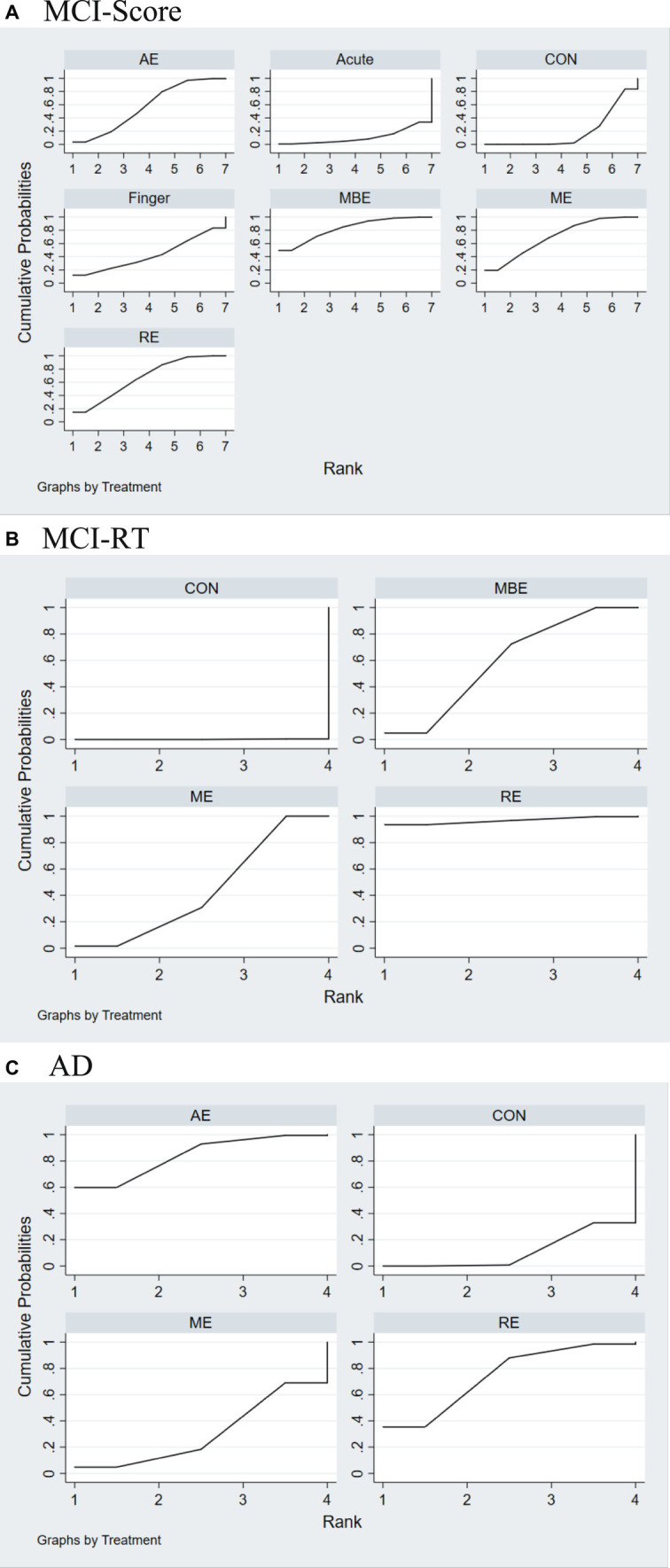
Results of the SUCRA. **(A)** MCI-Score. **(B)** MCI-RT. **(C)** AD. AE, aerobic exercise; ACUTE, acute exercise; CON, control group; FINGER, finger exercises; MBE, mind–body exercises; ME, multicomponent exercise; RE, resistance exercise.

In the reaction time group, the results of the inconsistency test demonstrated no inconsistencies among different types of physical exercise scores (*χ^2^* = 1.25, *p* = 0.264). The SUCRA ranking (Figure 4B) shows that RE (94.5) ranked the highest, followed by ME (58.1), AE (47.2), and finally CONTROL (0.3). The differences were statistically significant (*p* < 0.05). A comparison of the different exercise intervention groups and the control group revealed that all exercise interventions had a significant effect on VSWM of individuals with MCI, including RE (SMD = -36.91, 95% CI: −64.53 – -9.29), ME (SMD = -12.07, 95% CI: −14.62 – −9.52), and AE (SMD = -14.60, 95% CI: −22.94 – –6.25). The network plots are presented in Figures 4A,B. All exercise intervention groups displayed in the comparative form were directly compared with the non-exercise control group (Figures 5A,B). Table 6 presents the SUCRA results.

**Table 6:** Results of the SUCRA.

Treatment	SUCRA	PrBest	MeanRank
**A (MCI-Score)**			
MBE	82.9	49.6	2.0
ME	69.8	19.3	2.8
RE	67.2	14.8	3.0
AE	57.5	3.5	3.6
FINGER	42.8	12.1	4.4
CON	19.0	0.0	5.9
ACUTE	10.9	0.7	6.3
**B (MCI-RT)**			
RE	94.5	88.5	1.2
ME	58.1	9.0	2.3
AE	47.2	2.5	2.6
CON	0.3	0.0	4.0
**C (AD)**			
AE	84.1	59.9	1.5
RE	74.0	35.4	1.8
ME	30.7	4.8	3.1
CON	11.2	0.0	3.7

The MeanRank indicates the ranking with the highest probability for the corresponding technique, and SUCRA shows the probability of the technique that rank highest for each outcome; SUCRA, surface under the cumulative ranking curve; MCI, mild cognitive impairment; AD, Alzheimer’s disease; MBE, mind–body exercises; ME, multicomponent exercises; RE, resistance exercises; AE, aerobic exercises; FINGER, finger exercises; CON, control group; ACUTE, acute exercises.

### 3.6 Network meta-analysis results of AD studies

All types of exercise interventions produced superior outcomes to the non-exercise control group. The results of the inconsistency test showed no inconsistencies between the scores of the different types of physical activity (*χ^2^* = 3.40, *p* = 0. 182). The SUCRA ranking indicated that AE ranked best compared to the control group, with all three exercise interventions having higher impact effects than the control group; AE (84.1), RE (74.0), and ME (30.7) were higher than the CONTROL (11.2), and the differences were statistically significant (*p* < 0.05). The network plot is shown in Figure 4C. A comparison of the different exercise intervention groups with the control group demonstrated that all exercise interventions, except for ME, had a significant effect on the VSWM of individuals with MCI, including AE (SMD = 0.39, 95% CI: 0.06–0.71) and RE (SMD = 0.32, 95% CI: 0.02–0.61). The network plot is depicted in Figure 4C. All exercise intervention groups presented in the comparative form were directly compared with the non-exercise control group (Figure 5C).

## 4 Discussion

### 4.1 Summary of the evidence

**Figure 6:**
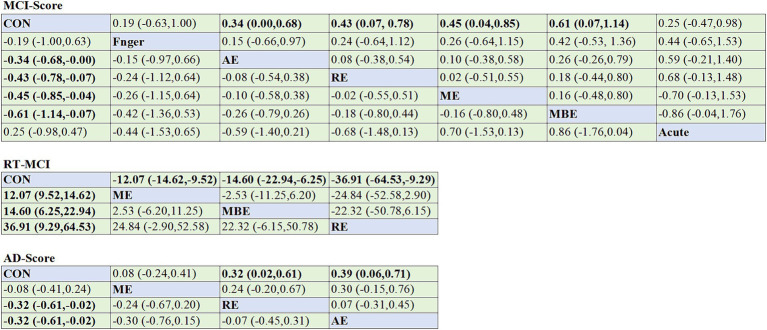
Comparative effectiveness results for MCI-Score **(A)**, MCI-RT **(B)**, and AD **(C)**. Each cell showed a 95%CI SMD. Important results are shown in bold. CI, confidence interval; AE, aerobic exercise; MBE, mind–body exercise; ME, multicomponent exercise; RE, resistance exercise; SMD, standardized mean difference.

This network meta-analysis explored how exercise therapies affected MCI and AD patients’ VSWM. Previous research only included traditional meta-analyzes comparing exercise regimens on healthy individuals’ VSWM, omitting ME.No publication bias was seen in MCI or AD research. In scores-based studies of MCI patients, MBE had the best results, followed by ME and RE. In reaction time experiments, ME, RE, and MBE were the most effective interventions. AD trials showed AE was most effective, followed by RE (Figure 6).

MBE, ME, RE, and AE were each found to enhance the VSWM of individuals with MCI. MBE incorporates a combination of physical exercise and various forms of cognitive training, such as social interaction or mental function exercises. AE is a component of MBE, and there is a connection between sustained AE and increased volume in both the left and right hippocampi of older adults (70). This suggests a correlation between AE and VSWM (71). The hippocampus is a brain region that is strongly associated with learning and memory; a larger hippocampal volume implies a greater number of neurons and synaptic connections that can effectively process and integrate VSWM information (72). Interventions that involve MBE and ME may have a stronger impact on the VSWM of individuals with MCI compared to singular forms such as AE and RE. This aligns with the viewpoint that an enriched cognitive environment may safeguard cognitive function in individuals with MCI (73). In an environment rich in multisensory feedback, this rich sensory stimulation facilitates greater neuronal signal transmission and synaptic plasticity (74).

Viewing reaction time as an outcome measure, MBE, RE, and ME improved the speed of individuals with MCI performing VSWM tasks. The impact of RE on the VSWM of individuals with MCI aligns with previous research showing that RE can strengthen connectivity in the networks of the temporal–parietal junction in the right hemisphere, the ventrolateral prefrontal cortex, and the dorsolateral prefrontal cortex, all of which are associated with response speed during cognitive tasks, in individuals with MCI (75). The improvement in the speed of VSWM tasks through ME and MBE in the MCI group is consistent with the results of a previous intervention study that focused on the working memory of women with MCI (76). Both ME and MBE emphasize integration between different components, mobilizing cognitive resources, and maintaining a focus on body awareness and action planning during the intervention process, ultimately leading to improved speed in VSWM tasks for individuals with MCI (77). Long-term engagement in MBE and ME provides a wide range of cognitive stimuli that contribute to the accumulation of greater cognitive reserve in individuals with MCI. These findings align with the cognitive reserve hypothesis (78).

AD research has mostly used scores to assess the effects of exercise therapy on VSWM tasks. I^2^ = 62.9% showed moderate heterogeneity in AD studies with no publication bias. Inconsistency tests revealed no substantial inconsistencies. Both AE and RE had significant intervention effects on the VSWM of individuals with AD, demonstrating the efficacy of AE and RE across different stages of cognitive impairment. However, ME did not improve the VSWM of individuals with AD, which is consistent with prior meta-analytic results (79). This could be attributed to the fact that ME interventions typically involve two or more types of exercises and multiple exercise tasks, potentially limiting the physical and cognitive abilities of individuals with AD and making it more challenging to achieve the optimal effects of a single-mode exercise intervention.

The impact of AE on the VSWM of individuals with AD can be explained as follows: AE can enhance neuronal activity and maintain normal functioning of the hippocampus. In addition, AE has the potential to reduce the rate of hippocampal atrophy in individuals with AD (80). The improvement in VSWM of individuals with AD achieved through RE may be associated with fat-free mass (FFM) of the limbs. A cross-sectional study involving 70 patients with AD who underwent magnetic resonance imaging scans found that the FFM of their limbs was positively correlated with their whole-brain volume (81). Additionally, in a study comparing FFM among individuals, those with lower FFM had a 1.43-fold higher probability of developing cognitive impairment than those with higher FFM (82). These findings suggest that strengthening FFM through RE can positively affect cognitive function. Our study also supports the feasibility of RE; however, further RCTs are required to elucidate the specific mechanisms underlying AD.

While APOE-4 was not specifically examined as a subgroup in our meta-analysis, it is important to highlight that there is a correlation between APOE and the efficacy of exercise intervention. APOE-4 is a significant genetic risk for Alzheimer’s disease (83). A research of 70-year-olds showed that individuals without APOE-4 benefit from physical activity in terms of protection against the disease, whereas those with APOE-4 do not (84). In a different cohort study with an average age of 50 years, carriers of the APOE-4 allele exhibited more robust protective effects from exercise compared to non-carriers (85). Neural damage accumulating in individuals with the APOE ε4 gene variant may be too severe to benefit from the therapeutic effects of physical exercise. Despite the presence of the APOE ε4 gene, individuals should still be encouraged to engage in physical activity due to its significant physical health advantages.

This subgroup analysis will help discover heterogeneity and allow for tailored exercise recommendations by comparing effect sizes across subgroups. A selective effect of single-mode intervention time, duration, intensity, and weekly frequency was found. Subgroup analysis of single-session intervention time in MCI trials showed no statistically significant effect size for interventions under 60 min. A significant impact size was usually detected when the intervention lasted beyond 60 min. The effect magnitude was greatest when the intervention lasted over 90 min (SMD = 0.875, 95% CI: 0.349–1.407). The study indicates that moderate exercise intensity significantly enhances VSWM in patients with MCI and AD. A recent study found that moderate to high-intensity exercise has similar beneficial effects on cognitive function in patients with cognitive impairment (86). A small number of high-intensity intervention experiments were used in our study, and all of the subjects were over 70 years old. This may be why the effect size of high-intensity exercise was not statistically significant. In AD research, an effective response is only evident when the intervention duration reaches 90 min or longer. Conversely, previous meta-analytic studies on the VSWM of healthy individuals have found that interventions lasting less than 60 min were effective (87). Our findings thus raise an intriguing question: Does greater cognitive impairment necessitate longer interventions to yield significant effects? This question warrants further investigation.

In addition to providing scientifically based exercise intervention programs, the willingness of cognitively impaired patients to participate is also crucial. Research has shown that exercise support from caregivers and social interaction with peers of similar cognitive levels can increase the exercise participation willingness of cognitively impaired patients (88). Additionally, the community environment can influence the exercise willingness of cognitively impaired patients. For instance, whether the community offers cognitively disabled patients exercise facilities, equipment, and specialist instruction and assistance.

Future research should expand this focus by investigating the VSWM of individuals with MCI across different age groups and by conducting long-term intervention studies on AD. These studies should involve continuous follow-up and provide detailed intervention protocols, including single-session intervention duration as well as intervention components, duration, and intensity. Comparing the differences between specific exercise programs is also highly necessary. Exploring the effects of more exercise programs on individuals with MCI and AD can help provide more effective and diverse exercise regimes. This study performed direct comparisons between the various intervention components. Although we gathered data from four direct comparisons, the number of intervention components was limited. Current research on VSWM is also fairly limited, with most studies failing to separately analyze data related to VSWM, instead incorporating working memory assessments. This approach is potentially problematic as it may yield inaccurate outcomes.

### 4.2 Limitations

This study has five limitations. Owing to the limited direct comparisons between different exercise interventions, our results are subject to increased uncertainty, and caution is warranted in interpreting the findings. Second, ME interventions do not have a standardized composition, with variations in the duration and intensity of each component. Hence, different ME regimens may have different effects. Future research may benefit from defining the specific components of multi-modal exercises and exploring the effect sizes associated with different multi-modal exercise approaches. Third, a wide variety of tools are available for assessing VSWM, with different measurement instruments exhibiting varying levels of reliability and validity. Due to the lack of race and gender comparisons in the previous literature, we did not conduct an analysis. However, there are certain racial disparities in cognitive impairment, and it is worth noting that there were more female participants in our sample. Therefore, discussing gender and race as variables is essential. These differences may lead to variations in effect sizes, necessitating careful consideration when selecting and interpreting the pooled effect sizes. Finally, this review primarily focused on older adults. As such, our conclusions are mostly applicable to older adults with MCI and AD. Future studies should include participants from different age groups to identify more comprehensive intervention strategies.

## 5 Conclusion

Our meta-analysis supports the notion that long-term physical exercise has a moderately beneficial effect on VSWM in individuals with MCI and AD, and it identifies optimal exercise intervention strategies applicable to these populations. Integrating the results of subgroup and network meta-analyzes, we recommend the following exercise regimens:

For individuals with MCI: Engage in moderate-intensity exercise(VO2max 50–75%) three to four times per week; interventions should exceed 60 min in duration and extend over a period of at least 3 months or longer; exercise modalities include MBE (e.g., Tai Chi, yoga), ME, RE (e.g., resistance band exercises, half squats, heel raises), and AE (e.g., indoor/outdoor walking, treadmill exercise, cycling training, stepping exercises). When opting for ME, we recommend incorporating aerobic exercise, resistance training, and balance training, while considering the individual’s physical condition. For individuals with AD: Engage in moderate-intensity exercise twice a week (VO2max 50–75%); interventions should exceed 90 min in duration and extend over a period of at least 3 months or longer; exercise modalities include AE and RE (same as mentioned in MCI). We also recommend that individuals with MCI and AD participate in exercise groups to enhance social interaction and increase motivation for exercise participation, thereby fostering adherence to long-term exercise.

Healthcare professionals selecting exercise programs should consider factors such as cost-effectiveness, safety, and the physical and social accessibility of required exercise facilities. Additionally, an assessment of individual physical condition and consideration of weather-related factors should be conducted in advance. In conclusion, our meta-analysis underscores the positive impact of exercise interventions on VSWM in individuals with MCI and AD.

## Data availability statement

The original contributions presented in the study are included in the article/supplementary material, further inquiries can be directed to the corresponding author.

## Author contributions

JD: Data curation, Formal analysis, Investigation, Visualization, Writing – original draft. HW: Supervision, Writing – review & editing. TF: Methodology, Writing – original draft. CX: Formal analysis, Writing – original draft. QZ: Investigation, Writing – review & editing. LG: Writing – review & editing. YZ: Conceptualization, Methodology, Supervision, Writing – review & editing.
